# Management of Dermatologic Events Associated With the Nectin-4-directed Antibody-Drug Conjugate Enfortumab Vedotin

**DOI:** 10.1093/oncolo/oyac001

**Published:** 2022-02-26

**Authors:** Mario E Lacouture, Anisha B Patel, Jonathan E Rosenberg, Peter H O’Donnell

**Affiliations:** Department of Medicine, Memorial Sloan Kettering Cancer Center, New York, NY, USA; Weill Cornell Medical Center, New York, NY, USA; Department of Dermatology, Internal Medicine, The University of Texas MD Anderson Cancer Center, Houston, TX, USA; Department of Medicine, Memorial Sloan Kettering Cancer Center, New York, NY, USA; Weill Cornell Medical Center, New York, NY, USA; Department of Medicine, Section of Hematology/Oncology, University of Chicago, Chicago, IL, USA

**Keywords:** enfortumab vedotin, skin reactions, anticancer therapy, Nectin-4

## Abstract

Enfortumab vedotin is a first-in-class Nectin-4-directed antibody-drug conjugate approved by the US Food and Drug Administration for the treatment of patients with locally advanced or metastatic urothelial cancer (la/mUC) previously treated with a platinum-based chemotherapy and a programmed death receptor-1/programmed death-ligand 1 (PD-1/L1) inhibitor, or patients with la/mUC who are ineligible for cisplatin-based chemotherapy and have previously received one or more prior lines of therapy. Enfortumab vedotin is the only drug to have demonstrated survival benefit versus chemotherapy in a randomized controlled trial in patients with la/mUC previously treated with platinum-based chemotherapy and a PD-1/L1 inhibitor. The development of dermatologic events following the administration of enfortumab vedotin is anticipated given the expression of Nectin-4 in epidermal keratinocytes and skin appendages (eg, sweat glands and hair follicles). There is the potential for rare but severe and possibly fatal cutaneous adverse reactions, including Stevens-Johnson syndrome and toxic epidermal necrosis, as described in the boxed warning of the US prescribing information for enfortumab vedotin. This manuscript describes the presumed pathophysiology and manifestations of dermatologic reactions related to enfortumab vedotin, and presents recommendations for prevention and treatment, to provide oncologists and other healthcare providers with an awareness of these potential adverse events to best anticipate and manage them.

## Teaser

Enfortumab vedotin is a Nectin-4-directed antibody-drug conjugate approved by the US FDA for the treatment of patients with locally advanced or metastatic urothelial cancer. This article describes dermatologic reactions related to enfortumab vedotin and presents prevention and treatment recommendations.

**Implications for Practice**

There is a need for efficacious and tolerable treatments for patients with locally advanced/metastatic urothelial carcinoma (la/mUC). Enfortumab vedotin is a first-in-class Nectin-4-directed antibody-drug conjugate for the treatment of la/mUC that has shown an overall survival benefit over standard-of-care chemotherapy. As Nectin-4 is expressed in epidermal keratinocytes and skin appendages, dermatologic events are common, anticipated treatment-related adverse events. Understanding the features of treatment-related dermatologic events and the prevention and management protocols should equip the healthcare team to educate, monitor, and manage patients who develop these reactions to minimize toxicity, maximize clinical benefit, and maintain quality of life.

## Introduction

Locally advanced or metastatic urothelial carcinoma (la/mUC) is an aggressive disease associated with a poor prognosis. The historical 5-year survival rate for metastatic disease in the US is 6.4%,^1^ demonstrating an unmet need for efficacious and tolerable treatments for patients with la/mUC. Only recently have developments such as immunotherapies and targeted therapies expanded treatment options and improved outcomes in patients with la/mUC. Dermatologic events are frequent, anticipated adverse events (AEs) associated with immunotherapies and targeted therapies, and require timely and appropriate management to maintain quality of life and continuation of therapy.^2-6^

**Figure 1.**
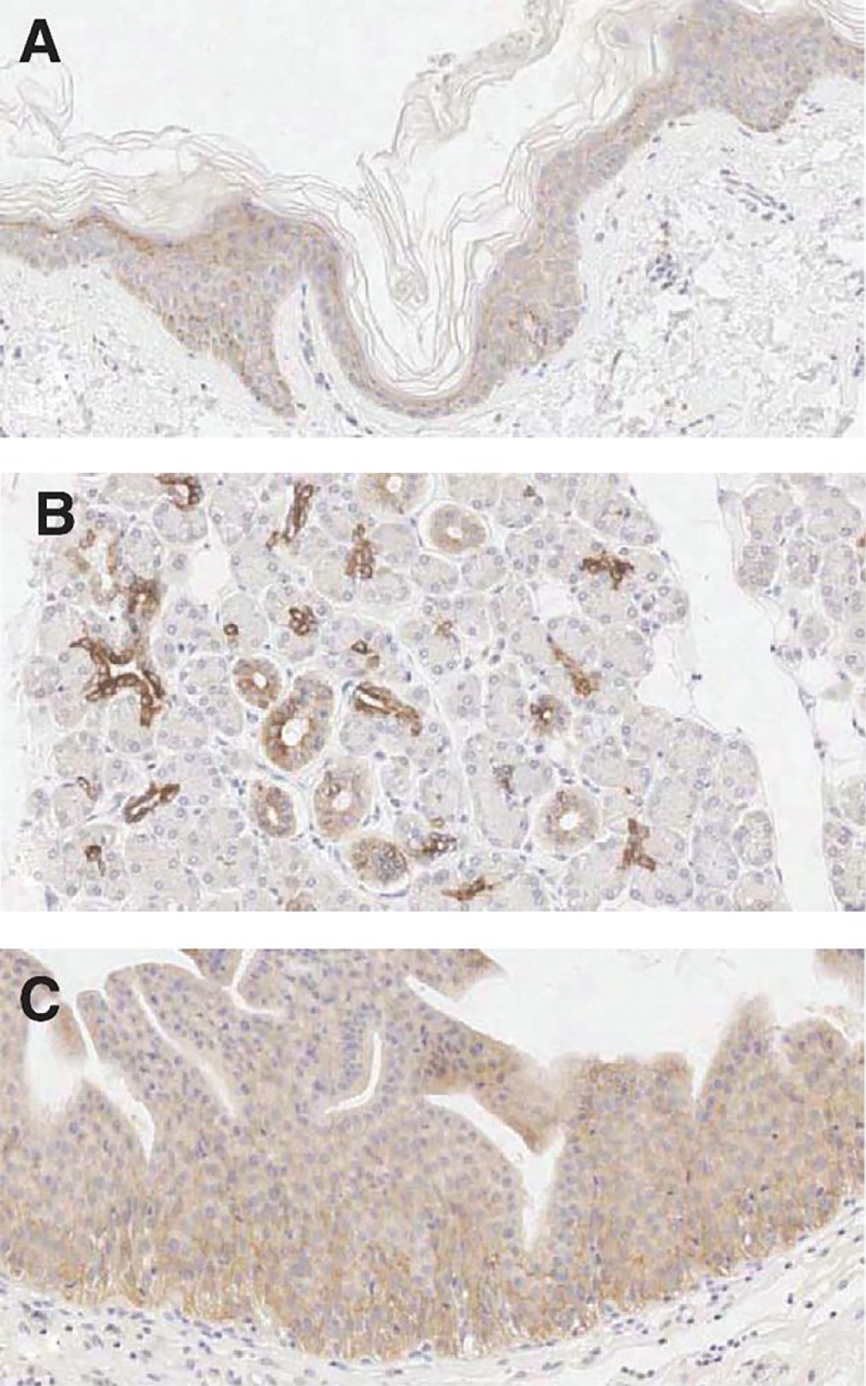
Positive staining for Nectin-4 human tissue sections is indicated by brown colored precipitate in skin (**A**), salivary glands (**B**), and bladder (**C**). Reprinted with permission from Challita-Eid PM, et al. *Cancer Res.* 2016;76:3003-3013.

One molecular target expressed in la/mUC is Nectin-4, a member of the family of immunoglobulin (Ig)-like adhesion molecules normally expressed in human epidermal keratinocytes and skin appendages (eg, sweat glands and hair follicles), as well as in the transitional epithelium of the bladder, salivary gland ducts, esophagus, breast, and stomach (Fig. 1).^7-10^ Nectins are thought to mediate cell-cell adhesions at adherens junctions, and to regulate several cellular activities, including cell motility, differentiation, polarization, proliferation, and survival.^11,12^ Nectin-4 is also highly expressed in urothelial as well as breast, ovarian, gastric, pancreatic, head/neck, esophageal, and lung carcinomas,^7,8,13-17^ and overexpression is associated with disease progression or poor survival in multiple tumor types,^7,15-22^ thus making Nectin-4 a molecular target with therapeutic potential.

Enfortumab vedotin is a first-in-class antibody-drug conjugate (ADC) consisting of a Nectin-4-directed, fully human IgG1 monoclonal antibody conjugated to monomethyl auristatin E (MMAE), a microtubule-disrupting agent, via a protease-cleavable linker.^7,23^ In the US, enfortumab vedotin is indicated for the treatment of patients with la/mUC previously treated with a platinum-based chemotherapy and a programmed death receptor-1/programmed death-ligand 1 (PD-1/L1) inhibitor, or patients with la/mUC ineligible for cisplatin-based chemotherapy who have previously received one or more prior lines of therapy.^24^ These indications are based on 2 global clinical trials evaluating enfortumab vedotin in patients with la/mUC: EV-201 (NCT03219333), a phase II, single-arm in patients previously treated with a PD-1/L1 inhibitor, and a platinum-based chemotherapy (Cohort 1) or were ineligible for cisplatin (Cohort 2),^13^ and EV-301 (NCT03474107), a randomized, open-label, phase III trial in patients with la/mUC previously treated with platinum-based chemotherapy and a PD-1/L1 inhibitor.^25^ In EV-301, enfortumab vedotin monotherapy versus chemotherapy (single-agent taxane [docetaxel or paclitaxel] or vinflunine) significantly improved overall survival (median overall survival: 12.88 vs 8.97 months; hazard ratio = 0.70, 95% confidence interval, 0.56-0.89; *P* = .001).^25^ The incidence of all-grade and grade 3 or higher treatment-related AEs was similar in the 2 treatment groups. The most common grade 3 or higher treatment-related AEs in the enfortumab vedotin arm included maculopapular rash, fatigue, and decreased neutrophil count compared with decreased neutrophil/white-cell count, anemia, and febrile neutropenia in the chemotherapy arm. Additionally, prespecified analyses of AEs of special interest (AESI) using composite categories of related AEs included any skin reaction (representing a set of dermatologic AEs), peripheral neuropathy, and hyperglycemia. In EV-301, all-grade treatment-related skin reactions, inclusive of all dermatologic AEs, occurred in 47% of patients who received enfortumab vedotin versus 15.8% of patients in the chemotherapy group. Skin reaction was the most common treatment-related AESI of grade 3 or higher in the enfortumab vedotin group, occurring in 14.5% of patients as compared to 0.7% in the chemotherapy group.^13,25^ Across enfortumab vedotin clinical trials, rare events of Stevens-Johnson syndrome (SJS) and toxic epidermal necrolysis (TEN) occurred and are included in the boxed warning of the US prescribing information for enfortumab vedotin; these events are described in detail later.

In this manuscript, we discuss the putative pathophysiology of enfortumab vedotin-related dermatologic events, varying clinical presentation, and potential predisposing risk factors. In addition, we describe recommendations for the prevention, monitoring, diagnosis, and management of these events, including when referral for specialized care is indicated.

## Pathophysiology and Clinical Presentation of Enfortumab Vedotin-Associated Dermatologic Events

**Figure 2.**
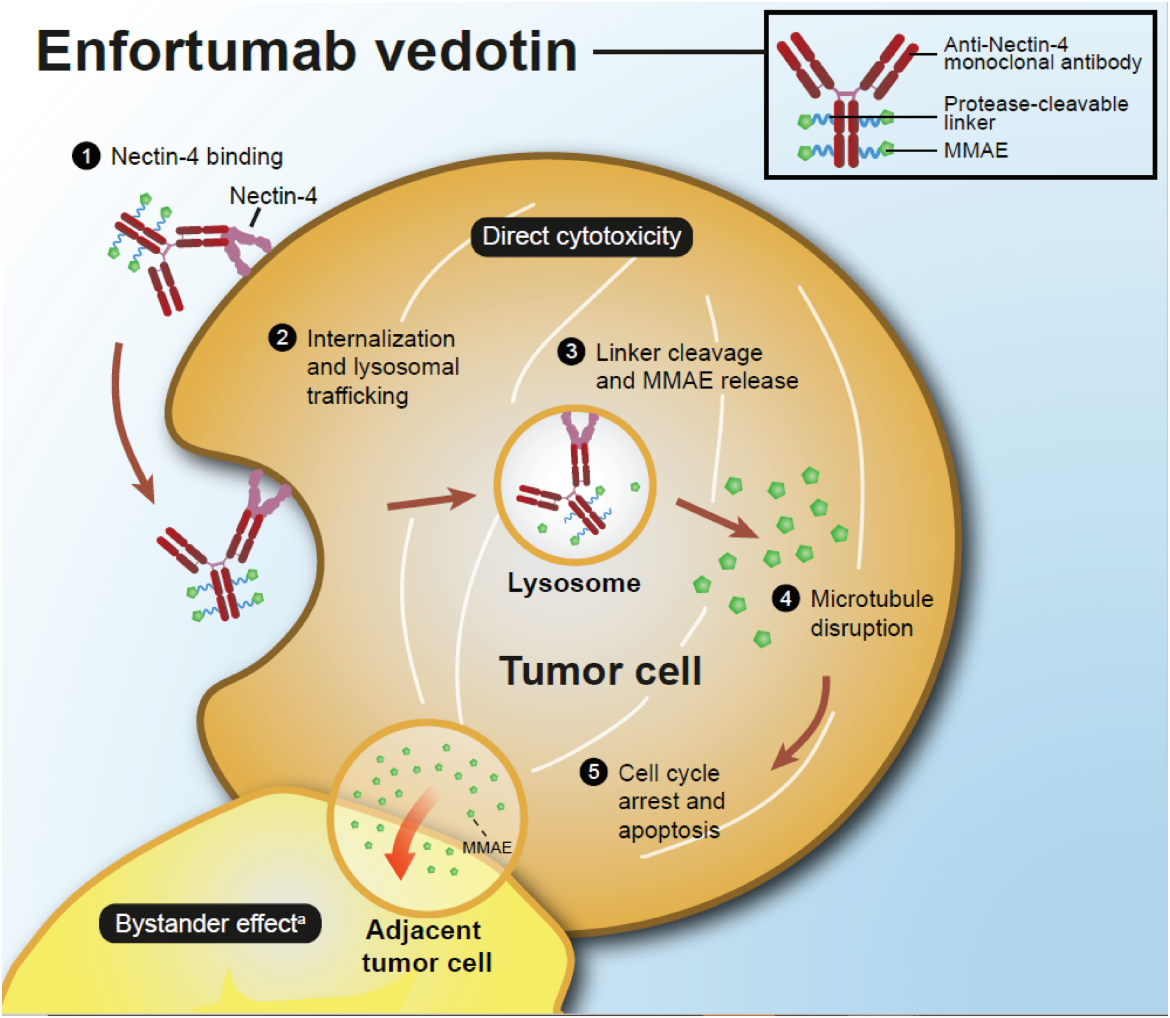
Enfortumab vedotin proposed mechanism of action.^7,26^^a^Additional mechanisms of action and their potential to complement the direct cytotoxicity of enfortumab vedotin are unknown.

**Figure 3.**
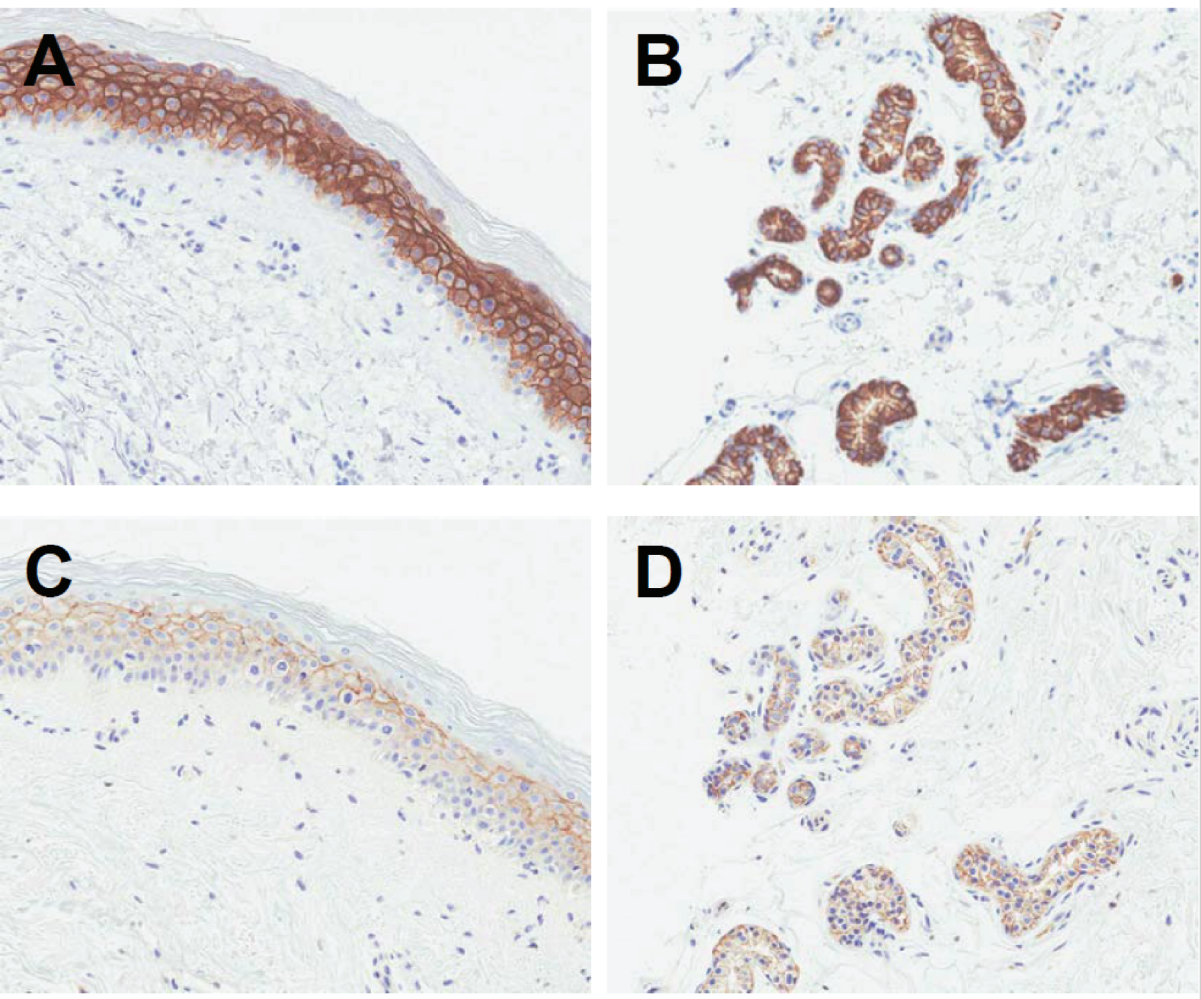
Expression of Nectin-4 in the skin. Positive Nectin-4 staining (brown) by immunohistochemistry is shown in patient skin biopsies of the epidermis (**A**) and sweat glands (**B**). In the same subject, enfortumab vedotin is shown to co-localize to these Nectin-4-positive tissues (brown staining in epidermis [**C**] and sweat glands [**D**]) consistent with the mechanism of action of enfortumab vedotin. The images were taken from a subject on Day 17 of Cycle 1. Data on file.

Enfortumab vedotin binds to Nectin-4 on the surface of cancer cells and causes direct cytotoxicity by inducing apoptosis. Once bound to the cell surface, enfortumab vedotin is internalized and MMAE is released into the cytoplasm. MMAE then binds and disrupts the microtubule network leading to cell cycle arrest and apoptosis (Fig. 2).^7,23,24^ Preclinical data suggest that in addition to direct cytotoxicity, enfortumab vedotin may have additional mechanisms of action including the bystander effect, which occurs when intracellular MMAE released from enfortumab vedotin diffuses across cell membranes and causes apoptosis in adjacent tumor cells.^26^ The mechanism by which enfortumab vedotin causes dermatologic events is presumed to be through the delivery of MMAE into Nectin-4-expressing normal tissue, such as the epidermis and epithelium of sweat glands and hair follicles (Fig. 3A,B). Specific binding of enfortumab vedotin to these skin structures was confirmed in a good laboratory practice (GLP) human tissue cross-reactivity study performed with the ADC (data on file). In phase I studies, enfortumab vedotin administered intravenously has been shown to localize to these tissue structures in patient skin biopsies collected up to 7 days post-treatment (data on file; Fig. 3C,D). Due to the mechanism of action of MMAE, actively dividing epidermal keratinocytes are particularly susceptible to the antimitotic effects of targeted MMAE delivery.

**Figure 4.**
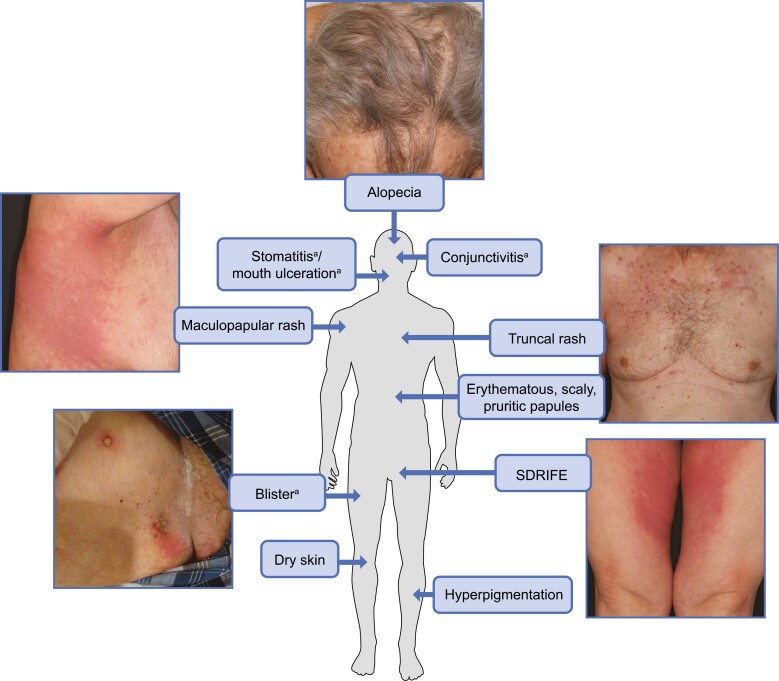
Schematic representation of selected dermatologic events associated with enfortumab vedotin. ^a^Included in composite term for severe cutaneous adverse reactions based on standardized Medical Dictionary for Regulatory Activities query. SDRIFE, symmetrical drug-related intertriginous and flexural exanthema. Photographs courtesy of Dr. Mario E. Lacouture.

The presentation of enfortumab vedotin-related dermatologic events varies in distribution (localized or widespread), morphology, symptomatology, and severity, with onset generally within the first cycle of treatment (Fig. 4).^13,24,25,27^ These events commonly present as erythematous, scaly, pruritic papules predominately in intertriginous, flexural, and acral areas with possible truncal involvement.^28-30^ Histopathology often demonstrates vacuolar interface dermatitis that may manifest clinically as erythema or hyperpigmentation.^29,30^ Other common histopathologic features include an intraepithelial ring or starburst mitotic features, interface dermatitis, and maturation disarray of epidermal keratinocytes.^29-31^ These inflammatory dermatologic events may impair the integrity and immune function of the epithelium, leading to an increased susceptibility to infection, transepidermal water loss, and symptoms such as dryness and pruritus.^13,25,29,30,32^

Dermatologic events commonly seen with enfortumab vedotin also occur with other targeted anticancer therapies as well as with immunotherapies.^4,6,33^ Such therapies, including enfortumab vedotin, may cause rare but potentially life-threatening skin reactions collectively known as severe cutaneous adverse reactions. Severe cutaneous adverse reactions are a subset of skin reactions that are predominantly caused by drugs and include SJS, TEN, symmetrical drug-related intertriginous and flexural exanthema (SDRIFE), drug reaction with eosinophilia and systemic symptoms (DRESS)/drug-induced hypersensitivity syndrome, and acute generalized exanthematous pustulosis (AGEP). These reactions can present with both dermatologic and non-dermatologic symptoms (ie, conjunctivitis, stomatitis, fever). The different types of severe cutaneous adverse reactions vary in terms of mortality rate, systemic involvement, and sequelae.^2,32,34^ For example, SDRIFE is distinct from other severe cutaneous adverse reactions due to its absence of systemic involvement. Although complications with SDRIFE are extremely rare given its self-limited course,^34^ more serious severe cutaneous adverse reactions must be ruled out to ensure proper management.^34,35^ SJS/TEN is associated with the highest mortality rate of all severe cutaneous adverse reactions.^32^ While the pathogenesis of severe cutaneous adverse reactions is not fully understood, they are thought to be Type IV delayed hypersensitivity reactions involving activation of CD8+ cytotoxic T cells and other immune cells, as well as secretion of cytokines and chemokines.^32,35,36^

## Dermatologic Events in Enfortumab Vedotin Clinical Trials

In the integrated safety population of 680 patients across enfortumab vedotin monotherapy trials, 55% of patients who received treatment at the approved dose of 1.25 mg/kg experienced skin reactions (as an AESI composite term encompassing the broadest capture of cutaneous events). The most common manifestations of skin reactions were maculopapular rash (23%) and pruritus (33%). Grade ≥3 skin reactions occurred in 13% of patients and included maculopapular rash, rash erythematous, rash or drug eruption, SDRIFE, dermatitis bullous, dermatitis exfoliative, and palmar-plantar erythrodysesthesia syndrome.^24^

In the enfortumab vedotin clinical trials, the median time to onset of the first observed skin reaction was within the first cycle.^13,25,37^ In these studies, severe cutaneous adverse reactions were reported as a composite term of dermatologic and non-dermatologic events because of potential ocular and mucosal involvement and included the terms “stomatitis,” “drug eruption,” “dermatitis bullous” or “blister,” “skin exfoliation,” “exfoliative rash,” “erythema multiforme,” “fixed eruption,” “pemphigus,” “toxic skin eruption,” “conjunctivitis,” and “mouth ulceration”. These events occurred in 20% of patients in EV-301.^13,25^ The incidence of enfortumab vedotin-related dermatologic events reported in the EV-301 clinical trial is summarized in Table 1.

**Table 1.** Enfortumab vedotin-related skin reactions reported in EV-301.

	EV-301N = 296n (%)	
	All grade	Grade 3/4
**Any skin reaction**	**139 (47)**	**43 (15)**
**Any severe cutaneous adverse reaction**^a^	**60 (20)**	**15 (5)**
**Preferred term**		
Rash maculopapular	48 (16)	22 (7)
Rash	45 (15)	5 (2)
Drug eruption^a^	26 (9)	8 (3)
Stomatitis^a^	21 (7)	1 (<1)
Conjunctivitis^a^	9 (3)	1 (<1)
Rash erythematous	8 (3)	4 (1)
Skin exfoliation^a^	7 (2)	1 (<1)
Dermatitis bullous^a^	6 (2)	2 (1)
Erythema	6 (2)	1 (<1)
Blister^a^	5 (2)	1 (<1)
Palmar-plantar erythrodysaesthesia syndrome	3 (1)	0
Eczema	3 (1)	0
Rash macular	2 (1)	0
Rash pruritic	2 (1)	0
Rash vesicular	1 (<1)	1 (<1)
Perivascular dermatitis	1 (<1)	1 (<1)
Toxic skin eruption^a^	1 (<1)	1 (<1)
Rash papular	1 (<1)	0
Erythema multiforme^a^	1 (<1)	0
Exfoliative rash^a^	1 (<1)	0
Dermatitis	1 (<1)	0
Fixed eruption^a^	1 (<1)	0
Pemphigus^a^	1 (<1)	0
Mouth ulceration^a^	0	0

a Severe cutaneous adverse reactions were reported as a composite term of dermatologic and non-dermatologic events for based on standardized Medical Dictionary for Regulatory Activities v23.0 query.

Data on file.

Treatment-emergent skin reactions were managed based on severity and associated symptoms. Mild skin reactions were treated with supportive care, including topical corticosteroids and oral antihistamines. For grade 3 events requiring intervention beyond supportive care, treatment with enfortumab vedotin was interrupted or delayed until improvement or resolution.^13,25^ Skin reactions led to discontinuation of treatment in 2.6% of patients in the integrated safety population (*n* = 680). Among the 59 patients in the integrated safety population who experienced a skin reaction necessitating dose interruption and who then restarted enfortumab vedotin treatment, 24% who restarted at the same dose and 16% who restarted at a reduced dose experienced recurrent severe skin reactions. Treatment-emergent skin reactions that resulted in dose reductions in EV-301 occurred in 8% of patients (Table 2).^24^

**Table 2.** Treatment-emergent skin reactions leading to dose modification or withdrawal in EV-301.

Preferred term	EV-301 N = 296, n (%)
**Skin reactions leading to dose reduction**	
Any skin reaction	24 (8)
Any severe cutaneous adverse reaction^a^	5 (2)
Rash maculopapular	13 (4)
Rash erythematous	1 (<1)
Rash vesicular	1 (<1)
Drug eruption^a^	4 (1)
Rash	3 (1)
Stomatitis^a^	1 (<1)
Eczema	1 (<1)
Erythema	1 (<1)
Perivascular dermatitis	1 (<1)
**Skin reactions leading to dose interruption**	
Any skin reaction	33 (11)
Any severe cutaneous adverse reaction^a^	14 (5)
Rash maculopapular	13 (4)
Rash	10 (3)
Rash erythematous	1 (<1)
Drug eruption^a^	7 (2)
Dermatitis bullous^a^	3 (1)
Dermatitis acneiform	2 (1)
Blister^a^	1 (<1)
Conjunctivitis^a^	1 (<1)
Fixed eruption^a^	1 (<1)
Skin exfoliation^a^	1 (<1)
Stomatitis^a^	1 (<1)
**Skin reactions leading to treatment withdrawal**	
Any skin reaction	12 (4)
Any severe cutaneous adverse reaction^a^	6 (2)
Rash maculopapular	4 (1)
Drug eruption^a^	2 (1)
Dermatitis bullous^a^	2 (1)
Rash	1 (<1)
Rash erythematous	1 (<1)
Conjunctivitis^a^	1 (<1)
Toxic skin eruption^a^	1 (<1)

a Severe cutaneous adverse reactions were reported as a composite term of dermatologic and non-dermatologic events for based on standardized Medical Dictionary for Regulatory Activities v23.0 query.

Data on file.

Across all studies of enfortumab vedotin monotherapy that included 749 patients (comprised of 680 patients with urothelial carcinoma and 69 patients with other tumor types), serious AEs of severe cutaneous adverse reaction were reported in 11 patients (1.5%) and included “dermatitis bullous” (0.4%), “drug eruption” (0.4%), “blister” (0.1%), “conjunctivitis” (0.1%), “SJS” (0.1%), “stomatitis” (0.1%), and “toxic skin eruption” (0.1%) (data on file).

Severe cutaneous adverse reactions have also been reported in the post-marketing setting. From the US Food and Drug Administration (FDA) approval of enfortumab vedotin in December 2019 through October 2020, 15 patients receiving enfortumab vedotin experienced severe cutaneous adverse reactions, some of whom had more than one event and some of whom died (data on file). Given the complexity and incomplete information associated with voluntary post-marketing reporting, the number of fatal outcomes caused by a severe cutaneous adverse reaction cannot be accurately determined. While post-marketing safety data cannot accurately estimate the frequency of these events or establish a causal relationship to drug exposure, it can provide insight into the nature of enfortumab vedotin-related dermatologic events.

## Diagnosis of Enfortumab Vedotin-Associated Dermatologic Events

There are currently no established risk factors for developing dermatologic events while on enfortumab vedotin treatment. General characteristics that may predispose patients receiving targeted anticancer treatment or immunotherapies to experience dermatologic events include prior cutaneous reactions to previous lines of anticancer therapies, including immunotherapies that can cause delayed immune-related AEs. Other predisposing factors include prior personal or family history of a dermatologic condition, allergic reactions, or xerosis; skin damage due to solar or therapeutic radiation; advanced age; and renal and/or hepatic impairment.^2,27,32,36,38^

Routine skin assessments starting with the initiation of enfortumab vedotin are important since dermatologic events typically occur within the first cycle, and frequent and thorough follow-up is key as the appearance of dermatologic events often evolves over time. Enfortumab vedotin-related dermatologic events tend to spare the face and are more typically seen in the intertriginous, flexural, acral, and truncal areas. The common progression observed clinically is erythema that then fades or darkens, often accompanied by desquamation as the reaction resolves. The patient and their caretakers should be informed that rashes and severe skin reactions, as well as ocular and mucosal symptoms, have occurred after administration of enfortumab vedotin, and that they should notify the treating clinician immediately of any new or worsening dermatologic events.

Severe cutaneous adverse reactions are frequently preceded by symptoms that can assist in early diagnosis, including malaise, fever ≥100.4°F, ocular and mucosal symptoms, and dermatodynia.^32^ Careful examination for mucosal involvement is important, as >90% of patients with SJS/TEN experience involvement of the oral, ocular, or genital mucosa.^39^ The appearance of these symptoms should be an indication for immediate evaluation and intervention, as early recognition and drug discontinuation are associated with better prognosis.^2,32^ Evaluation may include biopsy(ies) and close follow-up to assess the progression and evolution of the dermatologic event over time. A histological analysis demonstrating widespread full-thickness epidermal necrosis and detachment is suggestive of SJS/TEN and should prompt urgent referral for specialized care to confirm the diagnosis. It is critical that histologic findings are interpreted in conjunction with the full clinical picture and a thorough history. Major complications from secondary infections such as sepsis and cellulitis, long-term effects involving the skin and eyes, and the significant acute morbidity and mortality associated with SJS/TEN are further reasons why early diagnosis of these diseases is crucial.^2,32,40^

## Recommendations for Prevention and Management of Enfortumab Vedotin-Associated Dermatologic Events

The recommended dose of enfortumab vedotin is 1.25 mg/kg (up to a maximum dose of 125 mg) administered as an intravenous infusion over 30 minutes on Days 1, 8, and 15 of a 28-day cycle until disease progression or unacceptable toxicity. Starting with the first cycle and throughout treatment, patients should be closely and frequently monitored for dermatologic events.^24^

**Figure 5.**
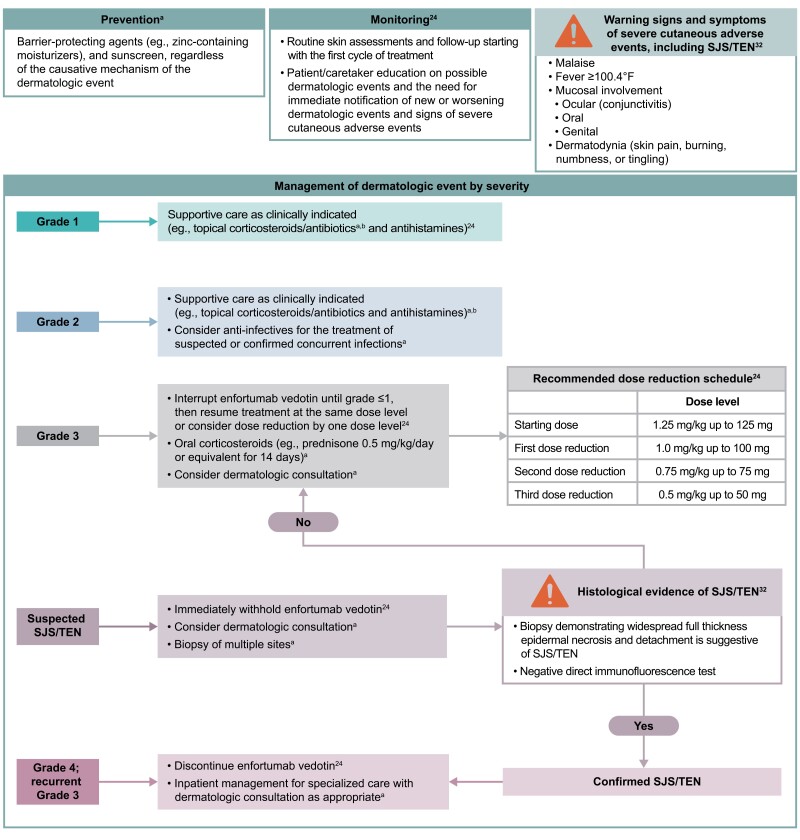
Recommendations for the prevention, monitoring, and management of enfortumab vedotin-associated dermatologic events. ^a^Recommendation based on clinical experience. ^b^Based on clinical experience, a combination of moderate potency topical corticosteroids and topical antibiotics (eg, triamcinolone 0.1% cream and silver sulfadiazine 1% cream) can be used. Topical treatment is usually required for at least 4 weeks, and once the event has resolved or decreased by one grade of severity, it may be utilized on an as-needed basis. Abbreviations: SJS, Stevens-Johnson syndrome; TEN, toxic epidermal necrolysis.

Prophylaxis protocols for treatment-associated dermatologic events are generally recommended as they may decrease the need for rescue therapies, lower the incidence of dose modifications, and improve quality of life.^41,42^ Clinical experience supports the prophylactic use of barrier-protecting agents such as zinc-containing moisturizers (eg, Desitin), especially in intertriginous areas like the axillae and groin, as well as ultraviolet radiation protection with sunscreen for photo-exposed areas (Fig. 5).^33,41^

Based on expert clinical recommendations, mild-to-moderate dermatologic events can be treated with moderate potency topical corticosteroids, combined with topical antibiotics for intertriginous areas (eg, triamcinolone 0.1% cream combined with silver sulfadiazine 1% cream) (Fig. 5). Topical treatment is usually required for at least 4 weeks, and once the event has resolved or decreased by one grade of severity, it may be utilized on an as-needed basis. There is also a potential role for systemic anti-infectives to be used as indicated for the treatment of concurrent bacterial or fungal infections.^33,39,43^

For severe (grade 3) dermatologic events, referral for specialized dermatologic care to assist in differential diagnosis and management should be considered. Enfortumab vedotin should be withheld until improvement to grade ≤1 (Fig. 5). Clinical experience has shown even brief dose holds (eg, 1 extra week “off” therapy at the end of a cycle) are effective and lead to resolution of the dermatologic event,^28^ likely due to the relatively short half-life of the ADC and MMAE (3.6 days and 2.6 days, respectively).^24^ Treatment can then be resumed at the same dose level, or with a dose reduction by one dose level at the discretion of the treating physician.^24^ Based on the clinical experience, oral corticosteroids (eg, prednisone 0.5 mg/kg/day for 14 days or equivalent) are advisable for grade 3 events. For cases of suspected SJS/TEN, treatment with enfortumab vedotin should be withheld immediately and management with dermatologic consultation should be considered.^24^ For grade 4 or recurrent (ie, 3 or more occurrences) grade 3 dermatologic events or confirmed SJS/TEN, treatment should be permanently discontinued,^24^ and admission to intensive care or burn unit may be warranted. The management of the ocular and mucosal symptoms that may be present in patients with SJS/TEN is beyond the scope of this manuscript; specialist consultation should be considered to manage these symptoms.

Recommendations for the management of AEs apart from the dermatologic events discussed here and also seen with other targeted therapies and immunotherapies have been detailed elsewhere.^4,6,27,33^

## Case Report

A 69-year-old man with metastatic urothelial cancer was commenced on enfortumab vedotin after progression of disease on prior immunotherapy. On Day 15 of Cycle 1 of enfortumab vedotin treatment, the patient developed pruritic, erythematous macules located in the folds of both elbows and the back of the neck. Topical diphenhydramine was effective in relieving the pruritus. Over the course of Cycles 2 and 3, the rash continued to progress despite the use of topical clobetasol, emollient and barrier treatment, and topical and systemic antihistamines, and the decision was made to withhold enfortumab vedotin for 3 weeks and re-evaluate.

At the next follow-up, the skin reaction had improved to grade 1, and Cycles 4 and 5 were administered at the full dose. Treatment was complicated by lower extremity cellulitis with blistering that responded to cephalexin. However, due to the worsening rash and the effect on the patient’s quality of life, several dose delays and treatment holds ranging from 1 week to 2 months were undertaken. The rash resolved completely after the prolonged 2-month treatment hold, and Cycle 6 of enfortumab vedotin was resumed at a reduced dose of 1 mg/kg.

Within 3 weeks of restarting enfortumab vedotin, a grade 2 pruritic maculopapular rash had again developed on the arms, abdomen, and legs of the patient, accompanied by sloughing of the skin on his legs, although the patient did not report any significant effect of the rash on his activities of daily living. He was using white petrolatum and clobetasol, both as needed. Additional cycles of enfortumab vedotin were marked by dose delays due to recurring rash and unrelated toxicities, including peripheral neuropathy. The patient achieved a complete response after 7 cycles of treatment before the patient ultimately discontinued treatment due to disease progression.

## Conclusions

Enfortumab vedotin monotherapy has demonstrated an overall survival benefit compared with chemotherapy in patients with la/mUC previously treated with platinum-based chemotherapy and a PD-1/L1 inhibitor. Enfortumab vedotin has also shown encouraging anti-tumor activity in patients with la/mUC ineligible for cisplatin therapy who have previously received one or more prior therapies. Enfortumab vedotin induces cell death through the preferential delivery of MMAE to Nectin-4-expressing cells. As Nectin-4 is normally expressed in epidermal keratinocytes and skin appendages, dermatologic events are common treatment-related AEs and should be anticipated by treating oncologists. Clinicians should monitor the patient frequently throughout treatment for the development of dermatologic events, starting early in the first cycle of treatment when the onset of these events typically occurs. Patients and caregivers should be educated on the signs and symptoms of dermatologic events and advised to report new or changing skin reactions to their treating clinician. Preventative strategies, including topical barrier-protecting agents, especially in intertriginous locations, and sunscreen in photo-exposed areas, are recommended. Mild-to-moderate dermatologic events can be managed with supportive care (eg, topical corticosteroids and antibiotics, antihistamines). For grade 3 dermatologic events, treatment with enfortumab vedotin should be withheld immediately, and referral for dermatologic consultation should be considered. Enfortumab vedotin should also be withheld for cases of suspected SJS/TEN and specialized care with dermatologic consultation should be considered. Treatment should be discontinued in any patients with confirmed SJS/TEN, any grade 4 skin reactions, or recurrent grade 3 dermatologic events. Monitoring for early signs of severe cutaneous adverse reactions is critical, as rare, fatal cases have been reported in clinical trials and in the post-marketing setting. By having an awareness of the pathophysiology of enfortumab vedotin-related dermatologic events as well as protocols for prevention and management, oncologists should be better equipped to educate, appropriately monitor, and manage patients who develop these reactions to minimize toxicity and maximize clinical benefit. The evaluation of the clinical activity and safety of enfortumab vedotin in earlier disease settings in urothelial cancer and in other solid tumors is currently ongoing.

## Data Availability

The data underlying this article will be shared on reasonable request to the corresponding author.
